# *Lnk/Sh2b3* deficiency restores hematopoietic stem cell function and genome integrity in *Fancd*2 deficient Fanconi anemia

**DOI:** 10.1038/s41467-018-06380-1

**Published:** 2018-09-25

**Authors:** Joanna Balcerek, Jing Jiang, Yang Li, Qinqin Jiang, Nicholas Holdreith, Brijendra Singh, Vemika Chandra, Kaosheng Lv, Jian-gang Ren, Krasimira Rozenova, Weihua Li, Roger A. Greenberg, Wei Tong

**Affiliations:** 1 Division of Hematology, Children′s Hospital of Philadelphia, Philadelphia, PA 19104 USA; 2 Department of Pediatrics, Perelman School of Medicine at the University of Pennsylvania, Philadelphia, PA 19104 USA; 3 Institute of Translational Medicine, School of Medicine, Yangzhou University, 225001 Yangzhou, Jiangsu China; 4 Department of Pathology & Laboratory Medicine, Perelman School of Medicine at the University of Pennsylvania, Philadelphia, PA 19104 USA; 5 Department of Cancer Biology, Abramson Cancer Research Institute and Basser Center for BRCA, and Perelman School of Medicine at the University of Pennsylvania, Philadelphia, PA 19104 USA

## Abstract

Fanconi anemia (FA) is a bone marrow failure (BMF) syndrome that arises from mutations in a network of FA genes essential for DNA interstrand crosslink (ICL) repair and replication stress tolerance. While allogeneic stem cell transplantation can replace defective HSCs, interventions to mitigate HSC defects in FA do not exist. Remarkably, we reveal here that *Lnk* (*Sh2b3*) deficiency restores HSC function in *Fancd2*^*−/−*^ mice. *Lnk* deficiency does not impact ICL repair, but instead stabilizes stalled replication forks in a manner, in part, dependent upon alleviating blocks to cytokine−mediated JAK2 signaling. *Lnk* deficiency restores proliferation and survival of *Fancd2*^*−/−*^ HSCs, while reducing replication stress and genomic instability. Furthermore, deletion of *LNK* in human FA-like HSCs promotes clonogenic growth. These findings highlight a new role for cytokine/JAK signaling in promoting replication fork stability, illuminate replication stress as a major underlying origin of BMF in FA, and have strong therapeutic implications.

## Abstract (web summary)

Loss of *Fancd2* leads to replication stress intolerance and Fanconi Anemia, where haematopoietic stem cell (HSC) function is compromised. Here, the authors show that *Lnk/Sh2b3* loss restores HSC proliferation and survival in *Fancd2* knockout mice and ameliorates replication stress in a cytokine/JAK2 signaling dependent manner.

## Introduction

Hematopoietic stem cells (HSCs) are characterized by their ability to self-renew and differentiate into multilineage blood cells^1,2^. They are the source of all circulating blood cells throughout life, and disruption of HSC homeostasis is associated with a variety of human disorders^1,2^. Faithful maintenance of genome integrity in hematopoietic stem and progenitor cell (HSPC) populations is crucial to hematopoiesis and suppression of blood-derived cancers. DNA repair deficiency, prominently illustrated by Fanconi anemia (FA) syndromes, results in progressive bone marrow failure (BMF) and cancer susceptibility^3,4^. Mutations within 22 genes have been identified as causative factors for FA. These genes cooperate in a genome stability network that is essential for repair of DNA interstrand crosslinks (ICLs) and relief of replication stress^5^. Cells derived from FA patients are hypersensitive to ICL-inducing agents mitomycin C (MMC) and cisplatin, and exhibit DNA damage checkpoint and mitosis defects^5^. Unrepaired DNA damage in HSPCs increases genome instability and leukemia/cancer in FA patients. The loss of HSPCs in FA is thought to be a consequence of multiple mechanisms, including impaired HSPC function, genotoxicity from the endogenous ICL agent, aldehyde^6,7^, physiological proliferative stress^8^, elevated p53 levels^9^, hypersensitivity to inflammatory cytokines^10,11^, oxidative stress^12^, and a hyperactive TGFbeta pathway^13^. However, how cellular signaling pathways converge on HSPC function to modify phenotypic outcomes in the context of FA mutations remains enigmatic. Importantly, other than allogeneic transplantation, therapeutic interventions that mitigate the HSPC defects in FA do not exist.

The FA pathway involves mono-ubiquitination of FANCD2-FANCI proteins by the FA core complex in addition to a parallel or downstream function of homologous recombination (HR) proteins, including the breast cancer suppressors BRCA1/2^14-17^. In addition to their established roles in repair of ICLs, the FA/BRCA protein network is activated by replication stress. This DNA repair-independent requirement for FA proteins, including FANCD2 and BRCA1/2, protects stalled replication forks from degradation^18,19^. These additional functions of the FA/BRCA proteins play a critical role in preventing genomic instability and suppressing tumorigenesis^18–20^. However, it remains to be determined how they are regulated by extracellular signals during physiologic hematopoiesis and whether stalled replication fork stability contributes to HSC attrition and BMF in vivo.

HSPC homeostasis is under the control of cytokine signaling pathways. One such important signaling axis is initiated by thrombopoietin (TPO) engagement of its cognate receptor, MPL to activate JAK2 tyrosine kinase signaling^21^. *Tpo*^*−/−*^ and *Mpl*^*−/−*^ mice exhibit marked reduction in HSC activity and HSC self-renewal^22,23^. *MPL* loss-of-function mutations are found in congenital amegakaryocytic thrombocytopenia (CAMT) patients, many of whom progress into BMF during childhood^24^. A critical negative regulator of the TPO/MPL pathway in HSCs is the adaptor protein LNK (or SH2B3)^25–27^. *Lnk* deficiency leads to a >10-fold increase in HSC numbers owing to superior HSC self-renewal^26,28^. Furthermore, *Lnk* deficiency strongly mitigates HSC aging and delays HSC exhaustion in serial transplants^29^. LNK directly interacts with phosphorylated JAK2 in a TPO-dependent manner, and *Lnk* deficiency potentiates JAK2 activation and signaling in HSPCs^25^.

In this study, we tested the hypothesis that *Lnk* deficiency would ameliorate HSC defects associated with FA. Remarkably, concomitant *Lnk* deficiency restored HSC function in *Fancd2*^*−/−*^ mice to wild-type levels without accelerating neoplastic transformation. Surprisingly, LNK did not overtly affect ICL repair in *Fancd2*^*−/−*^ cells but reduced spontaneous DNA damage and genome instability. This correlated with *Lnk* deficiency alleviating replication stress by stabilizing stalled replication forks in a manner dependent upon cytokine-mediated JAK2 signaling. Hence, these findings shed light on the underlying origin of BMF in FA patients and have implications for new therapeutic strategies.

## Results

### *Lnk* deficiency restores phenotypic HSCs in *Fancd2*^*−/−*^ mice

**Fig. 1:**
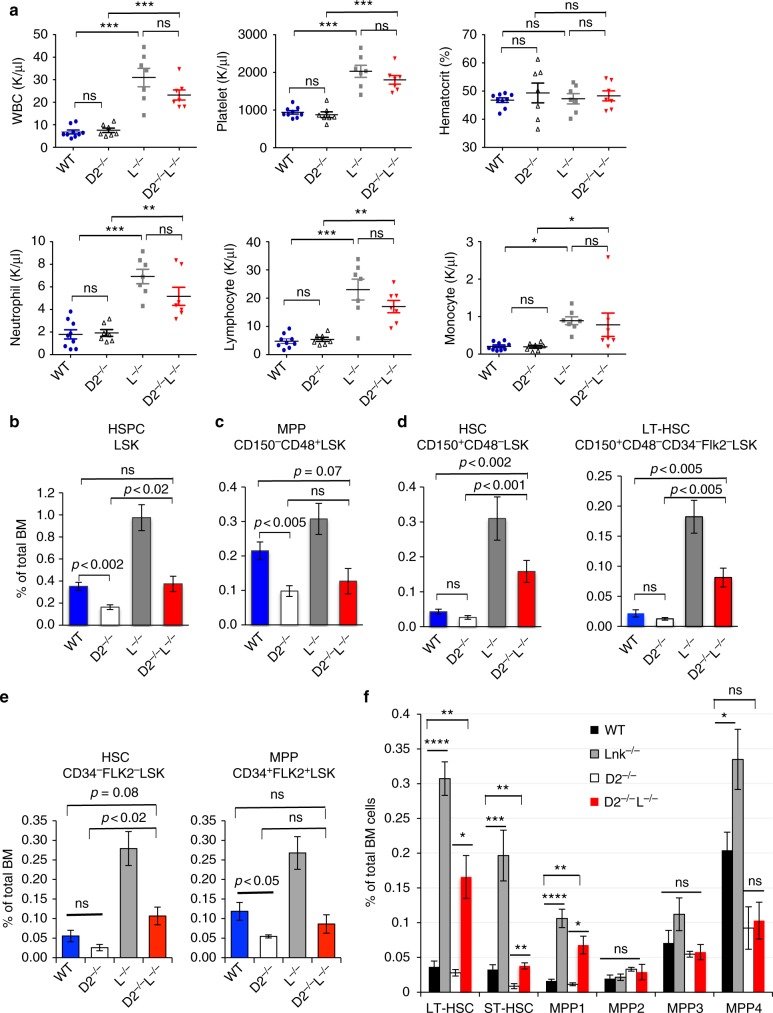
*Lnk* deficiency fully restores phenotypic HSCs in *Fancd2*^*−/−*^ mice. **a** CBC analysis of peripheral blood from WT, Fancd2^−/−^ (D2^−/−^), Lnk−/− (L^−/−^) and FancD2^−/−^*;*Lnk−/− (D2^−/−^L^−/−^) mice. WBC: white blood cell. Each symbol represents an individual mouse; horizontal lines indicate mean frequencies; error bars indicate SE. **b**–**d** Quantification of various HSPC compartments by flow cytometry using SLAM marker scheme, Lin-Kit + Sca1 + (LSK) HSPC population (**b**), MPP (CD48 + CD150-LSK) (**c**), HSC (CD48-CD150 + LSK), and LT-HSC (CD48-CD150 + Flk2-CD34-LSK) (**d**). **e** Quantification of various HSPC subsets using the CD34 and Flk2 surface marker scheme, HSCs (CD34-Flk2-LSK) and MPP (CD34 + Flk2 + LSK). **f** Quantification of various HSC and MPP subsets using indicated markers. LT-HSC: CD150 + CD48-Flk2-LSK; ST-HSC: CD150-CD48-Flk2-LSK; MPP1: CD150 + CD48-Flk2-CD34 + LSK; MPP2: CD150 + CD48 + Flk2-LSK; MPP3: CD150-CD48 + Flk2-LSK; MPP4: Flk2 + LSK. *P* values determined by two-tailed Student’s *t*-test are shown, *p < 0.05, **p < 0.01, ***p < 0.001, ns not significant. Data are pooled from 3–5 independent experiments

Mouse models of FA recapitulate cellular DNA repair defects and impaired HSPC function. To study if concomitant deletion of a negative regulator of cytokine signaling in HSCs, LNK, would restore HSPCs in mice deficient for Fancd2, a central component of the FA signaling pathway, we generated double nullizygous mice for *Fancd2* and *Lnk*. Complete blood counts revealed significantly elevated white blood cell and platelet numbers in *Lnk*^*−/−*^ mice, as previously reported^31,35^. *Fancd2*^*−/−*^ mice exhibited normal blood counts despite HSPC defects, consistent with previous reports^30^. Importantly, *Fancd2*^*−/−*^*;Lnk*^−/−^ mice showed similar blood counts to that of *Lnk*^*−/−*^ mice (Fig. 1a).

*Fancd2*^*−/−*^ mice are reported to have an ~50% reduction in HSPCs as indicated by Lineage^-^Kit^+^Sca1^+^ (LSK) cells^30,36^. However, the long-term (LT-) HSCs are only about 5% of the LSK fraction. To further pinpoint the defects in various HSPC compartments, we utilized a panel of cell surface markers to differentiate HSCs from multipotent progenitors (MPPs). *Fancd2*^*−/−*^ mice on a pure C57/B6J background exhibited a 50% reduction in MPPs while the phenotypic HSCs trended lower than wild type (WT) but this did not reach significance (Fig. 1b, c). Notably, loss of *Lnk* fully restored phenotypic HSCs in *Fancd2*^*−/−*^ mice as defined by SLAM markers (CD150^+^CD48^-^LSK)^37^ or CD34^−^Flk2^−^LSK^38^ markers, or the most stringent and current markers for LT-HSCs (CD150^+^CD48^−^CD34^−^Flk2^−^LSK)^39,40^ (Fig. 1d, e). In fact, *Fancd2*^*−/−*^*;Lnk*^−/−^ double mutant mice had more phenotypic HSCs than WT animals (Fig. 1b-e). We next analyzed subpopulations within the MPP compartments as defined previously^41,42^. *Lnk* deficiency markedly increased HSCs and primitive MPP (MPP1, CD34^+^Flk2^-^CD150^−^CD48^−^ LSK), but not MPP2 (Flk2^−^CD150^+^CD48^+^ LSK, megakaryocyte/erythroid-biased MPP) or MPP3 (Flk2^−^CD150^−^CD48^+^ LSK, myeloid-biased MPP), and slightly increased MPP4 (Flk2^+^CD150^−^ LSK, lymphoid-biased MPP) (Fig. 1f). *Fancd2*^*−/−*^*;Lnk*^*−/−*^ double mutant mice had more phenotypic HSC and MPP1 than WT animals, but no statistically significant difference in MPP2-4 (Fig. 1f). Thus, *Lnk* deficiency fully restores phenotypic HSCs and primitive MPP1 in *Fancd2*^*−/−*^ mice, but does not significantly alter lineage-biased MPPs.

### *Lnk* deficiency rescues *Fancd2*^*−/−*^ BM reconstitution ability

**Fig. 2:**
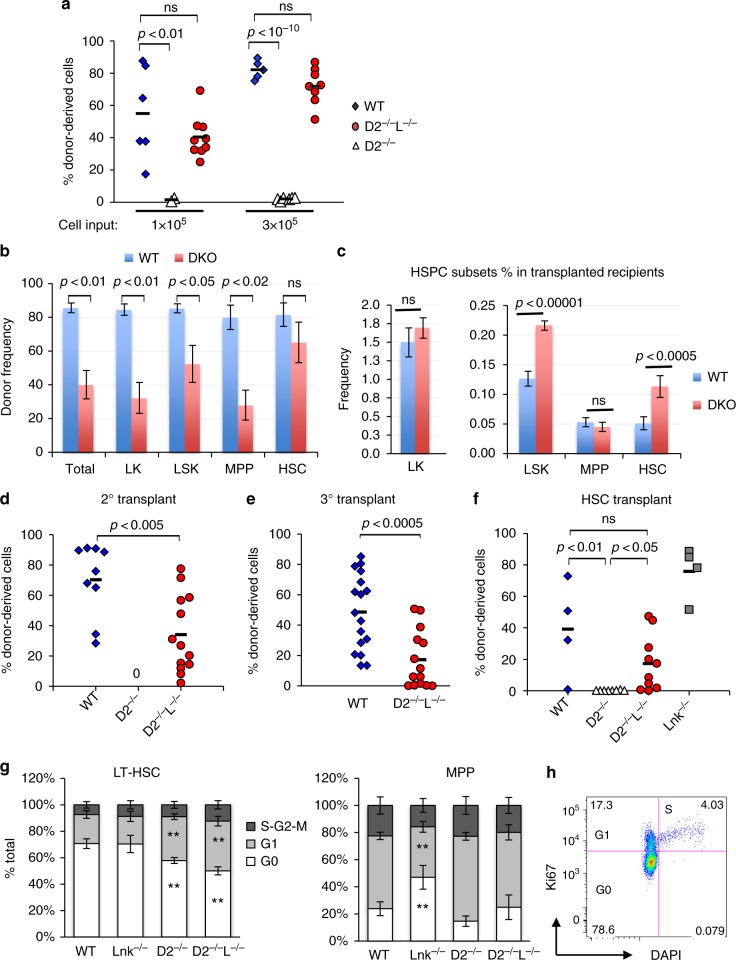
*Lnk* deficiency restores functional HSCs in *Fancd2*^*−/−*^ mice. **a**–**e** show serial transplantation of total bone marrow cells from WT, D2^−/−^ and D2^−/−^L^−/−^ mice. A graded number of BM cells were mixed with competitors and transplanted into lethally irradiated host animals. **a** Donor chimerisms in the peripheral blood of the recipient mice 16 weeks after transplant are shown. **b**–**c** At the end of primary transplant, donor frequencies (**b**) in the host BM as well as the frequencies of various HSPC populations (**c**) were quantified by flow cytometry. LK, Lin-Kit + ; LSK, Lin-Kit + Sca1 + ; MPP, CD150-CD48 + LSK; HSC, CD150 + CD48-LSK. **d**–**e** BM cells from the 3 × 10^5^ group were serially transplanted. Donor chimerisms in peripheral blood after secondary transplant (**d**) and tertiary transplant (**e**) are shown. **f** SLAM LSK HSCs were mixed with 3 × 10^5^ competitors and transplanted into lethally irradiated recipients. Donor chimerisms in peripheral blood 16 weeks after transplant are shown. Each symbol represents an individual mouse; horizontal lines indicate mean frequencies; error bars indicate SE. *p* values determined by two-tailed Student’s *t*-test are shown. ns: not significant. **g** shows quantifications of cell cycle analysis of LT-HSCs (Left) and MPPs (Right) in mice of different genotypes. **p < 0.01 compared to WT, two-tailed Student’s *t*-test. **h** shows a representative flow plot of cell cycle analysis in LT-HSCs. *P* values are calculated using two-tailed Student’s *t*-test in all plots. Data are pooled from 2–5 independent experiments

To assess if *Lnk* deficiency rescues the functional defects in *Fancd2*^*−/−*^ BM cells, we transplanted a graded number of unfractionated BM cells into lethally irradiated recipients. *Fancd2*^*−/−*^ BM cells showed markedly compromised reconstitution in bone marrow transplantation (BMT) assays (Fig. 2a) consistent with previous reports. Notably, the phenotypes we observed on the pure C57/B6 background were more severe than previous reports on mixed background^30,36^. Strikingly, the reconstituting ability of *Fancd2*^−/−^;*Lnk*^*−/−*^ BM cells was restored to WT levels (Fig. 2a). To test the self-renewal ability of HSCs, we performed serial BMTs. Although the donor percentage in the BM and various progenitor/MPP populations in *Fancd2*^*−/−*^*;Lnk*^*−/−*^ transplanted mice were inferior to WT transplanted mice at the end of primary transplant, donor percentage in the HSC compartment were on par with WT transplanted mice (Fig. 2b). Notably, there were more LSKs and HSCs in *Fancd2*^*−/−*^*;Lnk*^*−/−*^ transplanted mice than WT transplanted mice (Suppl. Figure 1), suggesting an overall increase in HSC reconstitution upon concomitant deletion of *Lnk* and *Fancd2*. As a result, *Lnk* deficiency largely restored HSC self-renewal in *Fancd2*^*−/−*^ BM cells in secondary and tertiary transplants, albeit slightly less than WT cells (Fig. 2c, d). *Fancd2*^*−/−*^ BM cells had near *zero* reconstitution in the transplants, underscoring the profound functional defects of FA cells as well as the significance of the rescue by *Lnk* deficiency. Of note, none of the serially transplanted mice developed leukemia.

### *Lnk* deficiency restores HSC functions in *Fancd2*^*−/−*^ mice

Since *Fancd2*^*−/−*^*;Lnk*^*−/−*^ double mutant mice have an increased number of phenotypic HSCs as assessed by cell surface markers, total BM transplants might reflect the number rather than the functionality of HSCs. Thus, we purified HSCs through Fluorescence-activated cytometric sorting (FACS) and injected equal numbers of HSCs into irradiated host animals. Our data showed that *Fancd2*^*−/−*^ HSCs also had a cell-intrinsic defect in reconstituting the hematopoietic system, while *Lnk* deficiency rescued the HSC functional defects associated with FA (Fig. 2e).

### *Lnk* deficiency does not restore *Fancd2*^*−/−*^ HSC quiescence

We reported previously that *Lnk*^*−/−*^ HSCs are more quiescent^25^, which protects them from regenerative stress. In contrast, FA-deficient HSCs show decreased quiescence and compromised self-renewal^30,36^. It is plausible that the rescued HSC compartment in double knockout (DKO) mice simply reflects an accumulating quiescent HSC population. To address this possibility, we quantified in vivo cell cycle kinetics in HSPC subsets. We found that *Fancd2*^*−/−*^*Lnk*^*−/−*^ HSCs were less quiescent than WT HSCs and showed a similar cell cycle profile to that of *Fancd2*^*−/−*^ HSCs (Fig. 2f, g). *Lnk*^*−/−*^ MPPs were significantly more quiescent than WT MPPs; however, *Fancd2*^*−/−*^*Lnk*^*−/−*^ MPPs showed a similar cell cycle profile to that of *Fancd2*^*−/−*^ MPPs (Fig. 2f, g). These data suggest that the restoration of *Fancd2*^*−/−*^ HSC activity to WT levels by *Lnk* deficiency is not be due to a restoration of HSC quiescence.

### *Lnk* deficiency does not rescue HSPC MMC hypersensitivity

**Fig. 3:**
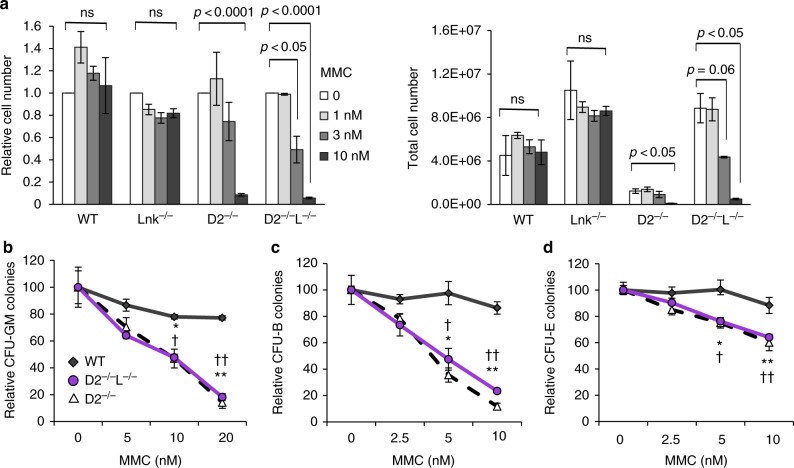
*Lnk* deficiency does not rescue ICL hypersensitivity associated with *Fancd2* deficiency. **a** HSPCs were cultured in a graded concentration of MMC for 7 days. Total (right) and relative to untreated cell number (left) are shown. Statistical analysis using two-tailed Student’s *t*-test of comparisons among untreated and MMC-treated cells within each genotype are indicated. **b**–**d** Fresh BM cells were plated in semi-solid methylcellulose media containing various concentrations of MMC and assessed for progenitors of myeloid (**b**), B cell (**c**), and erythroid lineages (**d**). Relative colony forming units relative to untreated are shown. *p < 0.05, **p < 0.01, two-tailed Student’s *t*-test, denotes comparison between WT and D2; † *p* < 0.05, †† *p* < 0.01, denotes WT vs DKO, at the corresponding dose of MMC. Representative data from three independent repeats are shown

The hallmark of FA in humans is the hypersensitivity to ICL-inducing agents. This feature is recapitulated in all FA mouse models. To explore the mechanisms by which *Lnk* deficiency ameliorates HSPC defects associated with FA, we first examined the MMC sensitivity of BM progenitor cells. Using both liquid culture growth assays and clonogenic survival assays, we found that both *Fancd2*^*−/−*^*Lnk*^*−/−*^ and *Fancd2*^*−/−*^ BM progenitors are strongly sensitive to MMC in comparison to WT progenitors (Fig. 3). *Lnk*^*−/−*^ single KO BM cells show similar MMC sensitivity to that of WT (Fig. 6b). Thus, *Lnk* deficiency does not appear to play an overt role in ICL repair.

### *Lnk* deficiency restores HSPC growth and genome stability

**Fig. 4:**
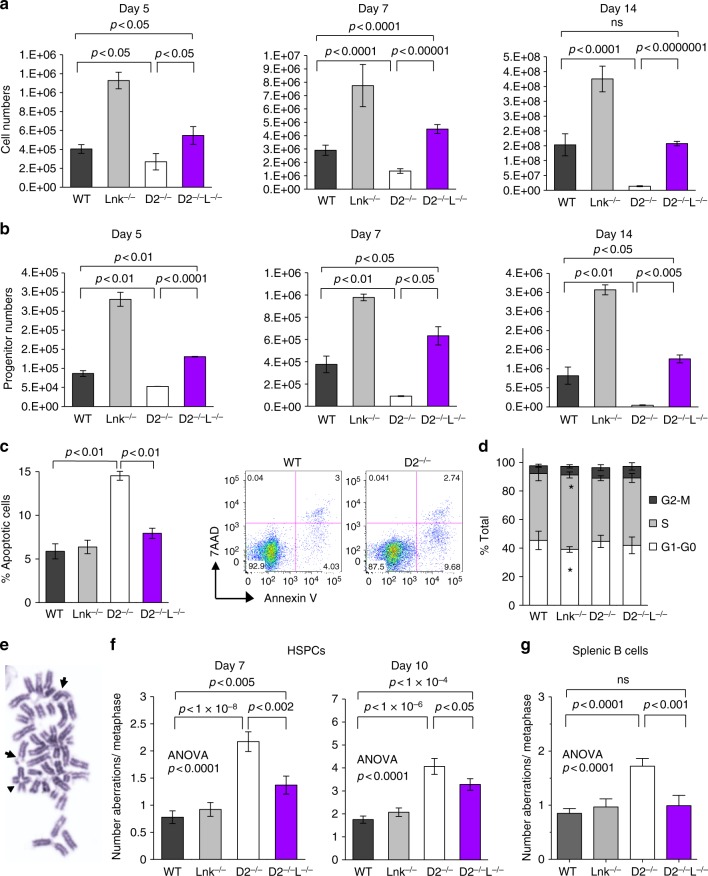
*Lnk* deficiency rescues the growth and survival defect of *Fancd2*^*−/−*^ HSPCs and mitigates genome instability. **a**, **b** HSPCs were cultured in TPO and SCF containing media for 14 days. One representative experiment of three independent replicates is shown. **a** Cumulative cell growth at days 5, 7, and 14 is shown. **b** At each corresponding day of culture, cells were plated for quantification of colony forming progenitors. Cumulative progenitor cell numbers were calculated and plotted. *P* values indicate two-tailed Student’s *t*-test. **c** On day 7, percentage of apoptotic cells was assessed by Annexin V + /7-AAD- cells and quantified. The right shows representative flow plots of Annexin V/7-AAD staining. **d** Quantification of cell cycle status in HSPCs using Ki67 and DAPI after 7 days in culture. Bars indicate mean and error bars indicate SE. *p < 0.05 compared to WT, Student’s *t*-test. **e**, **f** BM HSPCs cultured for 7 or 10 days in cytokines were assessed for chromosomal aberrations. **e** shows examples of chromosomal breaks (arrows) and radial chromosomes (arrowhead) scored on metaphase spreads. **g** Splenic B cells cultured for three days were assessed for chromosomal aberrations. **f**, **g** Quantifications of mean aberrations for 100–150 metaphase spreads from 4–6 animals are shown, and error bars indicate SE. Comparisons among all four genotypes were calculated by one-way ANOVA and are shown for each graph. *p* values using Tukey’s *t*-test for each indicated comparison pair are shown. Representative data from three independent repeats are shown

BM progenitors from BMF syndromes show growth retardation even in the absence of DNA damage-inducing reagents^43^. Thus, we subjected BM progenitor cells from *Fancd2*^*−/−*^ mice to liquid cultures in the presence of cytokines. Indeed, *Fancd2*^−/−^ HSPCs showed progressive loss of growth in comparison to that of WT or *Lnk*^*−/−*^ HSPCs (Fig. 4a). Importantly, *Lnk* deficiency rescued the growth disadvantage of *Fancd2*^*−/−*^ BM cells (Fig. 4a) as well as colony forming-unit (CFU) progenitors (Fig. 4b). FA cells are observed to have a higher rate of apoptosis^44^, consistent with our observations of a doubling in the percentage of apoptotic cells in *Fancd2*^*−/−*^ culture relative to WT (Fig. 4c). Despite these changes, the cell cycle progression remained essentially unperturbed in short-term cultures (Fig. 4d). Importantly, the percentage of apoptotic cells in DKO was markedly reduced in comparison to *Fancd2*^*−/−*^ cells (Fig. 4e), indicating that *Lnk* deficiency ameliorates the survival defect of FA.

The increased apoptosis in *Fancd2*^*−/−*^ cells may be attributable to a higher level of genome instability. Thus, we examined chromosomal abnormalities in ex vivo cultured progenitors from all genotypes. Metaphase spreads from nocodazole arrested BM HSPCs were prepared^45^. At least 30 metaphases from each sample were scored for the presence of sister chromatid breaks, gaps, and radial chromosome aberrations, which are hallmarks of ICL repair deficiency in FA. Chromosomal abnormalities were elevated in *Fancd2*^*−/−*^ cells as expected, which was partially suppressed by *Lnk* loss (Fig. 4e, f). Primary splenic B cells have been widely used to study molecular and cellular defects in FA mouse models^20^. To assess if *Lnk* deficiency restores genome instability in cell types other than BM HSPCs, we examined chromosomal aberrations in B cells, a cell type known to express *Lnk*. In agreement with genetic interactions in BM, genome instability in *Fancd2*^*−*^^*/−*^ B cells was also significantly elevated, while *Lnk* deficiency restored it to near WT levels (Fig. 4g). Together, these results reveal that *Lnk* deficiency suppresses proliferative and apoptotic abnormalities, while reducing genome instability in the *Fancd2*-deficient cells. Collectively, these data reveal genetic suppression of FA-mutation-associated hematopoietic phenotypes and genome instability by *Lnk* deficiency.

### *Lnk* deficiency restores HSPCs upon forced proliferation

**Fig. 5:**
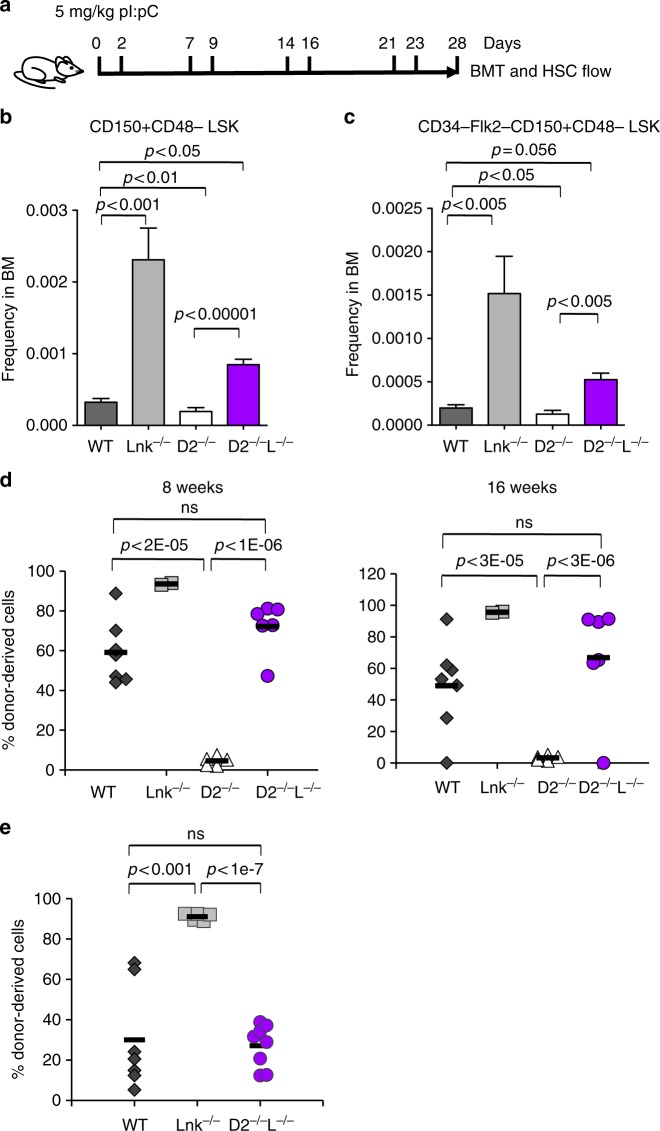
*Lnk* deficiency rescues HSC defects associated with FA upon forced in vivo proliferation. Proliferation of HSPCs in vivo was induced by pI:pC injection. **a** depicts a schematic overview of pI:pC injection. Mice were injected i.p. with 5 mg/kg pI:pC twice per week over four weeks, followed by analysis for HSPC frequency and function in bone marrow at day 28. **b**, **c **LT-HSC frequency determined by SLAM LSK (**b**) or CD34-Flk2-SLAM LSK (**c**) marker schemes is shown. Bars indicate mean of frequencies from four to six mice, and error bars indicate SE. Statistics were calculated by two-tailed Student’s *t*-test. **d** Two million total BM cells from pI:pC-treated mice were mixed with 3 × 10^5^ competitors and transplanted into lethally irradiated host animals. **e** In an independent experiment, one million total BM from pI:pC-treated mice were transplanted. **d**, **e** Donor chimerisms in peripheral blood of recipients 16 weeks after transplant are shown. Each symbol represents an individual recipient animal, horizontal bars represent the mean of each group. *p* values from two-tailed Student's *t*-test are shown. Two independent experiments are shown

FA cells are impaired by accumulated DNA damage from physiological stress during forced proliferation induced by transplantation or by administration of polyinosinic:polycytidylic acid (pI:pC)^8^. To determine whether *Lnk* deficiency rescues *Fancd2*^*−/−*^ HSPC function in context of endogenous replication stress, we induced HSPC proliferation in vivo by repeated treatment with pI:pC. We then examined phenotypic HSCs by FACS and HSC functions by BMT (Fig. 5a). Phenotypic HSCs were reduced in *Fancd2*^*−/−*^ mice relative to WT after pIpC stress, while HSCs remained expanded in *Lnk*^*−/−*^ mice (Fig. 5b, c). Importantly, *Lnk* deficiency restored HSC numbers in *Fancd2*^*−/−*^ mice (Fig. 5b, c). Functionally, *Fancd2*^*−/−*^ BM cells repopulated to a very limited extent upon stress (Fig. 5d, e). Remarkably, BMs from double nullizygous mice repopulated on par with WT in two independent experiments (Fig. 5d, e), indicating robust LT-HSC activity. Taken together, these data show that *Lnk* deficiency preserves HSC function in *Fancd2*^*−/−*^ mice upon physiological replication stress.

### *Lnk* deficiency stabilizes stalled replication forks in FA

**Fig. 6:**
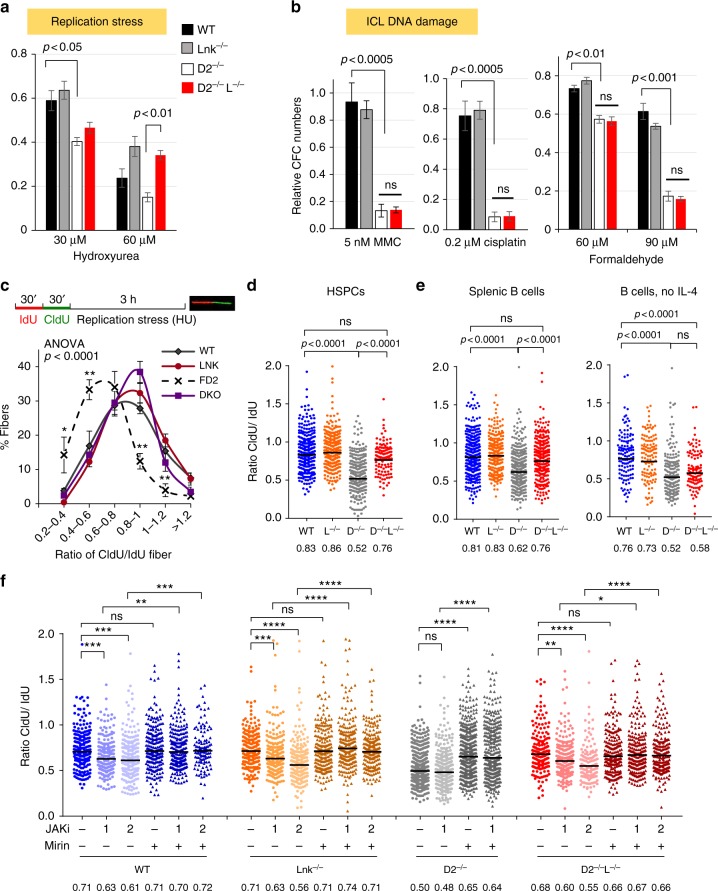
*Lnk* deficiency stabilizes stalled replication forks in *Fancd2*^*−/−*^ HSPCs through cytokine-JAK2 signaling. **a,** **b** BM cells from WT, D2^−/−^ and D2^−/−^L^−/−^ mice were plated in semi-solid methylcellulose media containing HU that induces replication stress (**a**) or indicated ICL DNA damage-inducing drugs, MMC, cisplatin, formaldehyde (**b**). Colony forming progenitor numbers relative to the vehicle-treated group (mean± SE) were enumerated and graphed. Representatives of three independent experiments are shown. Statistics were calculated using two-tailed Student’s *t*-test. (**c**). The top panel shows the experimental overview of the fork protection assay in single-molecule DNA fibers upon HU-mediated replication stalling. **c**, **d** Freshly isolated HSPCs (LSKs) from WT, D2^−/−^, *Lnk*^*−/−*^, and D2^−/−^Lnk^−/−^ (D2^−/−^L^−/−^) mice were subjected to fork protection assay. The frequencies of different replication tract ratios are plotted in (**c**). The distributions of CldU/IdU fiber ratios are shown in (**d**). **e** Splenic B cells were cultured in RP-105 and LPS with (Left) or without IL-4 (Right), then subjected to fork protection assay. (**f**) HSPCs from WT, D2^−/−^, and D2^−/−^L^−/−^ BMs were cultured in cytokines then subjected to the fork protection assay. 0, 1, or 2 µM JAK inhibitor ruxolitinib (JAKi) along with MRE11 inhibitor Mirin or vehicle alone were administered during the HU incubation in the fork protection assay. **d**–**f** The distribution of CldU/IdU fiber ratios is shown with the horizontal lines indicating geometric mean of fiber ratios, with corresponding number for each group shown at the bottom of the graph. **a–c** *P* values from two-tailed Student's *t*-test are shown. **d**–**f** Statistical significance of each set of conditions was calculated using Kruskal–Wallis ANOVA test and comparisons between individual groups were calculated using Dunn’s multiple comparison post test. *p < 0.05, **p < 0.01; ***p < 0.001; ****p < 0.0001; ns: not significant. Representative data from three independent repeats are shown

Cells deficient in FA/BRCA are sensitive to replication poisons, such as hydroxyurea (HU). To investigate a potential role of LNK in replication stress, we examined HU sensitivity of BM progenitor cells using clonogenic survival assays. *Fancd2*^*−/−*^ HSPCs showed a reduction in CFU progenitors upon HU treatment, and *Lnk* deficiency rescued progenitor clonogenic ability in *Fancd2*^*−/−*^ mice (Fig. 6a). In contrast, *Lnk* deficiency failed to rescue clonogenic ability of *Fancd2*^*−/−*^ HSPCs upon a number of ICL-inducing agents, including MMC, cisplatin, and formaldehyde (Fig. 6b). Thus, our data suggest that *Lnk* deficiency alleviates replication stress but not ICL-induced genotoxic stress associated with FA.

To examine the mechanisms by which *Lnk* loss ameliorates replication stress associated with FA, we treated BM progenitors with HU and examined the stabilization of stalled replication forks by single-molecule DNA fiber analysis as described^34^. Replication tracts of log-phase cultured HSPCs were sequentially pulse labeled with IdU and CldU before replication fork stalling by HU (Fig. 6c, d). Single DNA fibers were spread onto microscope slides before immunofluorescence staining with antibodies against IdU and CldU to measure relative fork length (CldU/IdU ratio). The relative shortening of the CIdU tract after HU treatment serves as a measure of replication fork degradation. WT and *Lnk*^*−/−*^ HSPCs cultured in cytokines showed a mean CIdU/IdU tract ratio close to 1 (Fig. 6c, d), indicating minimal replication fork degradation. However, *Fancd2*-deficient cells exhibited a significant reduction in the relative CIdU tract length as previously reported^18,19^ (Fig. 6c, d). Importantly, we discovered that loss of *Lnk* protected stalled replication fork from degradation upon replication stress in *Fancd2*^*−/−*^ progenitors as well as *Fancd2*^*−/−*^ B cells (Fig. 6c, d). This is in agreement with our result showing that *Lnk* deficiency conferred resistance to HU in clonogenic survival of HSPCs (Fig. 6a).

### Cytokine–JAK2 signaling stabilizes stalled replication fork

Since LNK negatively regulates the cytokine receptor-associated JAK2 kinase, we next asked whether its role in fork protection was cytokine signaling dependent. Since BM HSPCs depend upon cytokines for their survival in culture, we were unable to withdraw cytokines from BM culture. However, by removing IL-4 from the splenic B cell culture, it is possible to evaluate the role of cytokine-JAK signaling in isolation while still providing mitogenic stimuli to proliferating B cells through Toll-like receptor signaling. In these conditions, we found that *Lnk* deficiency restored replication fork protection in the presence of IL-4, but failed to rescue *Fancd2*- null B cells from replication fork degradation in the absence of IL-4 (Fig. 6e), suggesting that cytokine/JAK signaling plays a role in replication fork stabilization.

We next attempt to decipher the signaling pathways that contribute to replication fork stability in *Lnk* null HSPCs. LNK is a cytoplasmic adaptor protein that is not known to associate with chromatin. We previously reported that *Lnk* deficiency potentiates JAK2 activation in HSPCs, which in part accounts for its role in HSC self-renewal^25–27,31^. To define the role of JAK2 in replication fork stability, we subjected WT and *Lnk*^*−/−*^ HSPCs to JAK inhibitors (Ruxolitinib) and examined its effects on replication fork stability. We found that WT, *Lnk*^*−/−*^, and *Fancd2*^*−/−*^*Lnk*^*−/−*^ HSPCs were all sensitive to JAKi in stabilizing stalled replication fork (Fig. 6f). *Fancd2*^*−/−*^ HSPCs exhibited markedly reduced forks as previously reported but were not further shortened by JAKi (Fig. 6f). Importantly, the inhibitor Mirin prevented forks from degrading in all conditions, suggesting both JAKi conferred and FA-associated fork degradation were mediated by the nuclease MRE11 (Fig. 6f). Our data suggest that JAK signaling contributes to stalled replication fork stabilization in HSPCs through inhibiting MRE11-mediated nuclease activity.

It has been shown that the p53/p21 pathway is activated in FA cells and loss of p53 is able to partially restore the HSC defect in FA deficiency^9^. We thus examined p53 activation in the context of *Fancd2* and *Lnk* deficiency. By examining the expression of p53 target gene p21, we showed that p53 activation at steady-state in vivo was very low in *Fancd2*^*−/−*^ mice, in comparison to irradiation-induced acute p53 activation (Suppl. Figure 1), consistent with a recent report^46^. In this context, *Lnk* deficiency did not significantly reduce the modest p53 activation in *Fancd2*^*−/−*^ HSPCs (Suppl. Figure 1).

### *LNK* depletion restores FA-like human progenitor cell growth

**Fig. 7:**
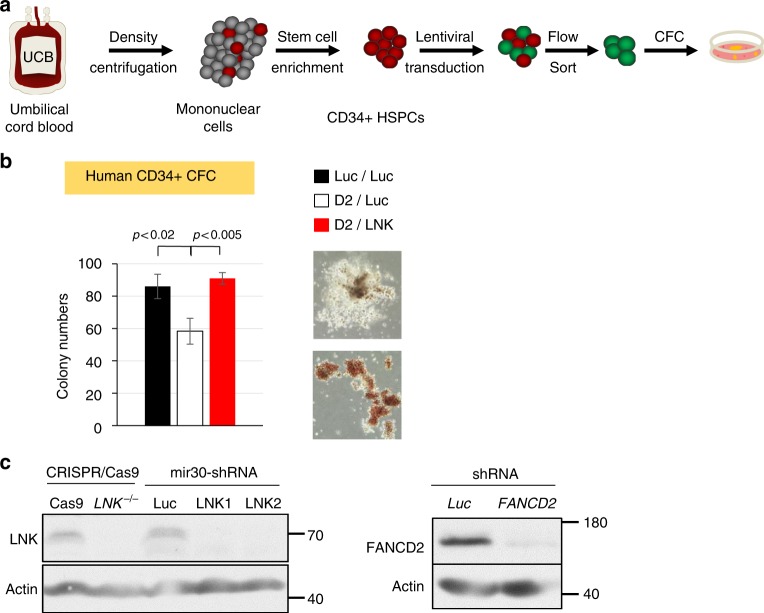
*LNK* depletion restores FA-like human progenitor cells. **a** depicts a schematic overview of isolation of primary human HSPCs for lentiviral transduction followed by CFC assay. **b** UCB-derived CD34 + cells were sequentially infected with lentiviruses expressing shRNA to Luciferase (Luc) or FANCD2 (D2) with GFP marker, followed by shRNA to Luc or LNK with mCherry marker. GFP + mCherry + cells were then sorted and plated onto semi-solid methylcellulose media. Colony forming progenitor numbers are shown with a two-tailed Student's *t*-test. Representative data from two independent repeats are shown. **c** TF-1 cells with shRNA-mediated depletion of *LNK* or *FANCD2* or CRISPR/Cas9-mediated depletion of *LNK* were analyzed for depletion efficiency by western blot. Luc, luciferase. gRNA, guide RNA

Our mouse genetic studies suggest Lnk as a potential target for increasing HSC number/function in the context of FA. Thus, we tested the effects of *LNK* deficiency in *FA*-like HSPCs, generated by knocking down *FANCD2* as previously reported in normal umbilical cord blood (UCB) HSPCs (Fig. 7)^9,33^. We showed that miR30-based shRNA knockdown of *LNK* exhibited high efficiency in depleting LNK proteins similarly to CRISPR/Cas9 (Fig. 7c)^47^. We then performed a proof-of-concept experiment in *FA*-like HSPCs generated by knocking down *FANCD2*, followed by *LNK* in human HSPCs. Our data showed that *LNK* depletion restored the growth and colony-formation ability of *FA*-like HSPCs **(**Fig. 7b**)**.

## Discussion

This report demonstrates that loss of *Lnk* restores FA HSPC functions in part by alleviating replication stress. These findings represent a rare in vivo example of genetic suppression of FA-associated HSC defects commensurate with restoration of genome stability. In contrast to *p53* loss, which restores hematopoietic function in FA mice concomitant with elevated genome instability and cancer, *Lnk* loss suppresses FA-associated HSC defects and increases genome integrity. Moreover, our results reveal a previously-unappreciated role for the JAK signaling pathway in promoting replication and genome stability. They are also suggestive that extracellular signals could be important modifiers of intrinsic deficits present in HSC from FA patients and other syndromes stemming from replication stress. To our knowledge, such a phenotypic reversal has not been reported for the canonical FA pathway. These results have several parallels to suppression of *BRCA1* null phenotypes by concomitant loss of *53BP*1^48–50^, which restored in vivo viability while reducing genomic instability and cancer. Very few animal models exhibit elevated HSC numbers and function^51^. Understanding how *Lnk* deficiency imparts this remarkable enhancement in HSC fitness will likely advance our understanding of stem cell biology and offer insights into strategies for the treatment of FA and other BMF diseases in general.

Lnk deficiency increased phenotypic and functional HSCs in *Fancd2*^*−/−*^ mice as assessed by numerous independent parameters. HSC activity in reconstituting lethally irradiated recipient mice was largely restored to WT level in the absence of *Lnk* and *Fancd2*. Moreover, *Lnk* deficiency mitigated HSC exhaustion upon physiological stress caused by repeated pIpC treatment. The ex vivo results were also consistent with the in vivo BMT data, as reconstitution of myelo-ablated animals is a form of “forced” proliferation. *Lnk* deficiency did not protect HSC quiescence in FA, rather it allowed *Fancd2*^*−/−*^ HSCs to proliferate and self-renew as a consequence of reducing replication stress. Previous work demonstrated that p53/p21 activation contributes to the HSPC decline and BMF in FA^99^. However, p53/p21 upregulation observed in freshly- isolated *Fancd2*^*−/−*^ HSPCs were not changed in cells with concomitant loss of *Lnk*, suggesting that the rescue of FA stem cells by *Lnk* deficiency at the steady-state in vivo cannot be attributed to p53 inhibition. Nonetheless, it is possible that *Lnk* deficiency reduces p53/p21 induction and/or other p53 regulated genes in the stress conditions. While it has been previously demonstrated that the survival of *Fancd2*^*−/−*^ cells can be improved by inactivation of apoptotic signaling with deletion of *p53*^9^ or inflammatory signaling with inactivation of TNFα signaling^52^, the genomic instability in *Fancd2*^*−/−*^ cells was not demonstrably suppressed in these scenarios. In contrast to the aforementioned modifying pathways, *Lnk* deficiency mitigates replication stress-incurred chromosomal aberrations in FA mice and prevents HSPCs attrition without further accelerating leukemic transformation.

Our study raises the question of what causes the underlying stem cell attrition and BMF in FA patients. It has been suggested that endogenous aldehydes, in the forms of acetaldehydes from cellular lipid peroxidation and formaldehydes from histone, and nucleic acid demethylation, lead to ICL DNA damage^6,53^. Indeed, FA patients and mice are more sensitive to deficiencies in the aldehyde detoxification enzymes *Aldh2* and *Adh5*^6,53^. Our findings show that *Lnk* deficiency protects stalled replication forks from degradation and stabilizes genome integrity of *Fancd2*^*−/−*^ HSPCs without restoring ICL-induced DNA repair. Therefore, replication stress may also be an important mechanism for HSC attrition and BMF in FA. *Lnk* deficiency increases stem cell fitness and alleviates HU-incurred replication stress. This translates to a superior HSC self-renewal and serial transplantability upon forced proliferation in vivo or in vitro. We speculate this DNA repair-independent mechanism is an important cause of HSC exhaustion in FA, which is illustrated by the rescue of *Lnk* deficiency in mitigating replication stress. Interestingly, relief of replication stress rather than restoration of DNA repair has also been invoked as the basis for genetic suppression of *BRCA* null phenotypes^20,54^. Although our data suggest that LNK does not have an overt role in ICL repair, it does not exclude a role for LNK in alternate DNA repair pathways. The downstream effectors of FA proteins, BRCA1,2 and RAD51, play critical roles in HR, which contributes to FA pathogenesis and genome stability. *Lnk* deficiency could increase replication-associated HR, as HR plays a crucial role in the survival of BRCA2 deficient mammary epithelial cells^55^. Moreover, TPO was shown to increase DNA repair efficiency in HSCs through an enhanced non-homologous end joining (NHEJ) mechanism upon irradiation^56^. Thus, increased NHEJ and/or HR in the absence of *Lnk* may help *Fancd2*^*−/−*^ HSC survival. Future efforts are warranted to formally test if LNK plays a role in other types of DNA repair in physiologically relevant cells. How cytokine receptor signaling affects DNA repair is not understood and this has implications for genome integrity in cancer and response to chemotherapy for FA.

LNK is a cytoplasmic adaptor protein, which is not known to associate with chromatin. A recent large-scale proteome effort identified hundreds of proteins enriched in nascent chromatin^57^. However, LNK was not among them and has not been implicated in functioning within the nucleus. Given its role in limiting cytokine signaling, we hypothesize that LNK regulates the activity of kinase cascades that transmit their signals to the nucleus to affect the expression or activity of DNA replication and repair proteins. Indeed, a role for JAK2 in replication and HR has been suggested. Overexpression and activation of WT JAK2 increases both RAD51 foci formation and HR activity without affecting NHEJ^58^. The oncogenic JAK2 mutant (*V617F*) found in myeloproliferative neoplasm (MPNs) is associated with increased DNA damage and replication stress^58,59^; however, JAK2^V617F+^ MPN patients typically remain clinically and cytogenetically stable over decades^60^, perhaps through upregulation of RECQL5^61^. *Lnk* deficiency potentiates JAK2 activation; however, it does not result in constitutive activation of JAK2 or cytokine-independent growth in HSPCs^25^. This important distinction between *Lnk* deficiency and the JAK2^V617F^ oncogenic mutant provides a probable explanation for their distinct consequences on replication stress. Our results highlight the importance of cytokine-mediated JAK2 signaling in ameliorating replication stress in primary HSPCs. This highlights a new role for cytokine/JAK signaling in promoting replication fork stability, along with its established role in signaling transcription. Future efforts are warranted to elucidate the mechanisms by which LNK-regulated JAK signaling functions in replication stress alleviation. Taken together, our data suggest that replication stress mitigation, but not ICL repair, contributes to this remarkable rescue of FA stem cells by *Lnk* deficiency. It is appreciated that LNK-regulated signaling pathways may play pleiotropic roles in FA stem cells, including stalled replication fork protection, oxidative stress mitigation, and reduction of replication-associated DNA damage as well as p53 induction, all of which could contribute to the impact of LNK in HSC fitness and suppression of FA.

**Fig. 8:**
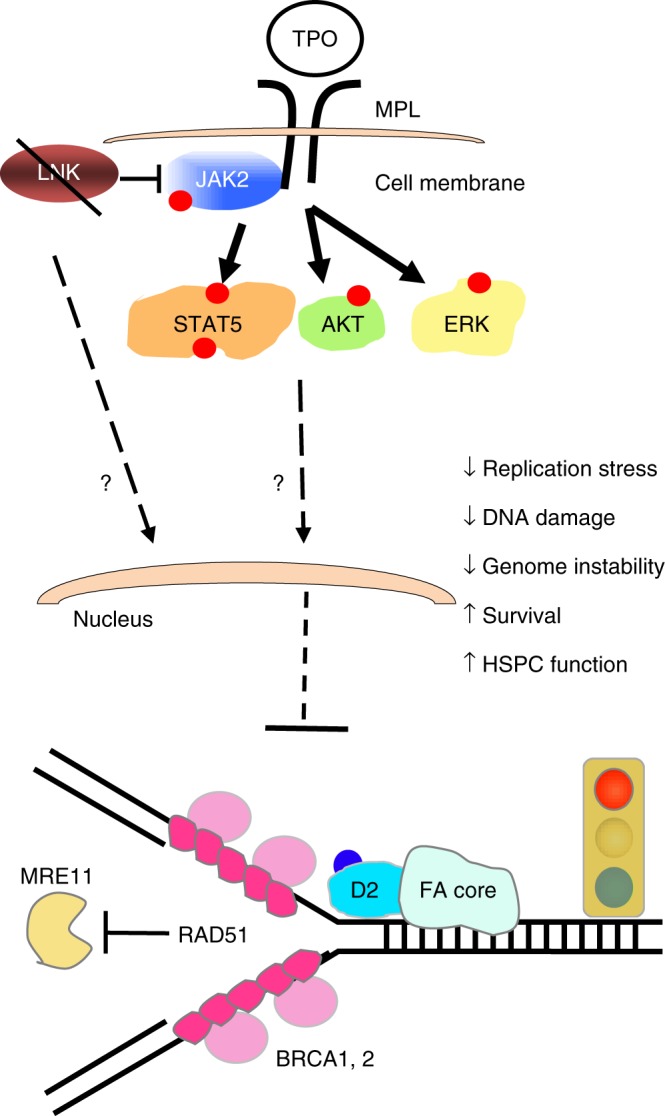
Working model of how LNK signaling regulates replication stress and restores HSPC function in FA

Notably, TPO agonist Eltrombopag showed encouraging clinical results in treating aplastic anemia and restores trilineage hematopoiesis in refractory severe aplastic anemia^62,63^. Our data on the TPO/JAK2 regulator LNK suggest that Eltrombopag might be an effective therapeutic for FA patients. However, we caution that TPO alone may not be sufficient to restore hematopoiesis or prevent HSC attrition in human FA. It is noted that TPO was included in our ex vivo HSPC culture, but it was not sufficient to mitigate genome instability, protect stalled replication fork, or prevent apoptosis in *Fancd2*^*−/−*^ murine HSPCs. Thus, we propose in a model (Fig. 8) that LNK exerts its function in a TPO/MPL/JAK2-dependent and independent manner. Of great clinical relevance, we provide a proof-of-concept experiment showing that deletion of *LNK* in human FA-like HSPCs promoted clonogenic growth. Thus, our results suggest a future therapeutic use of *LNK* inactivation to expand human HSPCs from FA patients.

## Methods

### Mice

*Fancd2*^*−/−*^ mice were generously provided by Dr. Alan D’Andrea (Dana Farber Cancer Institute) 30 and *Lnk*^*−/−*^ mice by Dr. Tony Pawson 31 (Samuel Lunenfeld Research Institute, Canada), respectively. All mice were on C57/B6J background (CD45.2). Transplant competitor cells were from SJL (CD45.1) mice and transplant recipients were progenies of SJL and C57/B6J (F1). Mice 2–6 months old of both sexes were used in the studies. The protocol (#2016–7–781) for this work is approved by Institutional Animal Care and Use committee (IACUC) of Children’s Hospital of Philadelphia (CHOP).

### Constructs

The miR30-based shRNA targeting human *LNK* (CAAAGATGATGTCTGTCCG) was constructed into the lentiviral vector (pCL20.MSCV.mir30.PGK.mCherry) generously provided by Dr. Shannon McKinney-Freeman^32^. The pTRIP-MND-GFP lentiviral vector containing shRNA to *FANCD2* was generously provided by Drs. Ceccaldi and Soulier^9,33^. ShRNAs targeting Luciferase were used as controls in both vectors.

### Western blot analysis

For western blot (WB) analysis, cells were directly lysed in in LDS reagent (Invitrogen), lysates resolved on Tris-Glycine polyacrylamide gels (National Diagnostics) and transferred to nitrocellulose membranes (Protran). The WBs were carried out with the following antibodies: LNK (rabbit anti human LNK peptide antibody, 1:1000), FANCD2 (rabbit anti- human FNACD2, Novus Biologicals #NB100–182, 1:1000) and Actin (goat anti- Actin Santa Cruz #sc-1616, clone I-19, 1:1000). Secondary antibodies conjugated to horseradish peroxidase against rabbit or goat IgG were used (Jackson Immunoresearch, #711–035–152 and #305–035–045, respectively, 1:20,000) followed by detection with Western Lightning chemiluminescent reagent (Perkin Elmer). Uncropped scans are shown in Supplemental Fig. 2.

### Primary human CD34 + HSPC cell isolation/ transduction

De-identified UCB was obtained from New York Blood Center. CD34 + HSPCs were isolated by density gradient centrifugation (Lymphoprep, SepMate-50 tubes, Stemcell Tech), followed by magnetic separation on an AutoMACS Pro using microbeads conjugated with anti-CD34 antibodies (Miltenyi). CD34 + cells were cultured overnight in StemSpan SFEM II (Stemcell Tech.) supplemented with 10% fetal bovine serum, penicillin/streptomycin, L-glutamine, 2-mercaptoethanol, 1 μM SR-1 (Stemcell Tech.), 100 ng/mL SCF, 40 ng/mL FLT3L, 50 ng/mL TPO, 20 ng/mL IL3, 20 ng/mL IL6, and 15 ng/mL GM-CSF (Peprotech Inc). Subsequently, cells were sequentially transduced by spinoculation at 1500 RPM at 37 C for 90 min on retronectin-coated plates loaded with pTRIP-MND-GFP (first transduction) or pCL20.MSCV.mir30.PGK.mCherry (second transduction) lentiviral particles in culture media in the presence of 10 μg/mL polybrene. Subsequently, mCherry + GFP + cells were sorted and plated onto semi-solid methylcellulose media (Methocult H4230, Stemcell Tech) supplemented with 5 U/mL EPO, 10 ng/mL IL-3, 5 ng/mL SCF, 5 ng/mL GM-CSF. Colonies were enumerated 12–14 days after plating.

### Antibodies

Antibodies used for HSC FACS and sorting were: Lineage (biotin-conjugated anti-Gr-1 (RB6–8C5), -Mac1 (M1/70), -B220 (RA3–6B2), -CD19 (eBio1D3), -Ter119 (TER-119), -CD5 (53–7.3), -CD4 (GK1.5), -CD8 (53–6.7) at 1:40 dilution), along with APC-Cy7-c-Kit (2B8), PerCP-cy5.5-Sca1 (E13–161.7 or D7), FITC -CD48 (HM48–1), PE-Cy7-CD150 (TC15–12F12.2), APC -CD34 (RAM34), and PE–Flk2 (A2F10.1), at 1:200–1:400 dilutions. Peripheral blood was analyzed using 1:200 dilution of anti PE-Cy7-CD45.1 (A20), APC-Cy7-CD45.2 (104), APC -CD19 (eBio1D3), PE -CD3e (145–2C11), or APC -Gr-1 (RB6–8C5), and PE–Mac1 (M1/70). FACS antibodies were purchased from eBioscience, BD Biosciences or BioLegend. BrdU analog CldU was detected by anti-BrdU (Abcam BU1/75, ab6326, 1:100 dilution) and AF488 anti rat antibodies (Invitrogen A-11006, 1:200), and IdU by anti-BrdU (BD B44, 347580, 1:100) and AF568 anti mouse antibodies (Invitrogen, A-11031, 1:200).

### Hematology, flow cytometry and cell sorting

Complete blood count was measured using a Hemavet 950 (Drew Scientific). For flow analysis of peripheral blood, red blood cells were first lysed then stained for donor reconstitution (CD45.1, CD45.2) in myeloid (Gr-1, Mac1) or lymphoid (CD19, CD3) lineages (all used as 1:200 dilution). All peripheral blood data was acquired using the BD Canto flow cytometer. For bone marrow analysis of transplanted mice, cells were stained with APC-Cy7-conjugated anti-CD45.1, Lineage (biotin-Ter-119, -Mac-1, -Gr-1, -CD4, -CD8α, -CD5, -CD19 and -B220) followed by staining with streptavidin-PE-TexasRed (Invitrogen SA1017, 1:50), and the HSPC panel: -c-Kit-APC, -Sca1-PE, -CD150-PE-Cy7, -CD48-FITC (1:200 dilution). Data for bone marrow analysis was collected on the BD Fortessa flow cytometer. For HSPC subset analysis, kit-APC-Cy7, Sca1-PerCP-Cy5.5, CD150-PE-Cy7, -CD48-FITC, CD34-APC, and Flk2-PE antibodies (1:200) were added to the FACS panel. All flow cytometry data was analyzed using FlowJo v8.7 for MAC.

For sorting, HSPCs were pre-enriched using a lineage depletion kit with magnetic separation (Miltenyi). Lin- cells were stained with the HSPC panel. One hundred LT-HSCs (SLAM LSK) were sorted using a drop envelope of 1.25 using a BD Aria sorter into individual wells of a 96-well plate containing 100 µL of StemSpan SFEM (STEMCELL) with 2% fetal bovine serum (SAFC Biosciences). Competitor cells were added directly to each well in 50 µL of PBS with 2% fetal bovine serum, then transplanted. HSPCs (LSK) for in vitro assays were Lin-depleted and sorted on the MoFlo Astrios EQ using the “purify” setting.

### Bone marrow transplant

Total bone marrow or sorted LT-HSCs (CD150 + CD48-c-kit + Sca1 + Lin-) from donor mice were mixed with 3 × 10^5^ freshly isolated competitor (B6.SJL) cells and injected retro-orbitally into lethally irradiated (10 Gy, split dose, Orthovoltage Precision X-Ray) F1 recipient mice. At 4, 8, 12, and 16 weeks post transplantation, peripheral blood of recipients was analyzed for donor reconstitution in myeloid, T- and B- lineages by flow cytometry. At 16 weeks post transplant, recipients were sacrificed and donor reconstitution in various HPSC compartments was analyzed using flow cytometry. BM cells from primary transplants were harvested and 2 million cells were injected into each secondary recipient. Tertiary transplants were similarly performed.

### pI:pC

Mice were injected with 5 mg/kg pI:pC (InvivoGen) i.p. twice weekly for 4 weeks. On day 28, BM cells were isolated and HSCs were quantified by FACS. One or two million total bone marrow cells from pI:pC-treated mice were transplanted into each lethally irradiated recipients.

### Colony forming assay

Total BM or sorted LSK cells were plated onto M3434 semi-solid methylcellulose media (STEMCELL Technologies). Plates were seeded in triplicate in the absence or presence of different drugs. Colonies were enumerated 7–12 days after plating.

### Liquid culture of primary HSPCs and splenic B cells

Sorted LSK were cultured in 96-well plates containing 100 µL StemSpan supplemented with 10% FBS and 1% penicillin/streptomycin, 1% L-glutamine, 100 µM β-mercaptoethanol (Sigma), 10 ng/mL TPO, and 50 ng/mL SCF (Peprotech Inc), with or without MMC. Cells were seeded at a density of 100,000/mL in triplicate and maintained at less than 1 million/mL throughout culture by subculture into fresh media. Viable cells were counted using a Hemacytometer (Hausser Scientific) using Trypan Blue (STEMCELL technologies).

Splenic B cells were cultured in 6-well plates containing 2.5 mL RPMI (Invitrogen) supplemented with 10% FBS, 1% penicillin/streptomycin, 1% nonessential amino acids (Life Technologies), 1% sodium pyruvate (Life Technologies), 50 µM β-mercaptoethanol, 5 ng/mL murine IL-4 (Life Technologies), 0.5 µg/mL RP105 (BD Pharmingen, 552128, 1:100), and 25 µg/mL LPS (Sigma).

### Cell cycle and apoptosis assays

Cell cycle analysis was performed using a BrdU kit (BD Biosciences, 552598). Cells were exposed to BrdU for 30 min and BrdU incorporation was assessed according to manufacturer’s instructions. For analysis of apoptosis, cells were incubated with anti-Annexin V antibody (Biolegend, 556420, 1:100) in Annexin V binding buffer (BD Biosciences) for 15 min. Cells were then stained with 7-AAD (BD Biosciences). Both BrdU and Annexin V staining data were acquired using a BD Canto flow cytometer.

Cell cycle analysis on HSPCs from BM was performed with freshly isolated HSPCs. Lin-depleted BM cells were stained for HSPC markers, then fixed and permeabilized followed by staining with anti-Ki-67-APC antibodies (eBio 50–5698–80). Cell cycle analysis is performed along with DAPI staining for DNA content and assessed on a BD Fortessa flow cytometer.

### Metaphase spreads

Splenic B cells or LSKs were cultured for 3 or 7 days, respectively, and then treated with 0.5 µM Nocodazole (Calbiochem) for 3 h to arrest in metaphase. Cells were incubated in 75 mM potassium chloride (Sigma) at 37 °C for 20 min to swell cell volume, and subsequently fixed with 3:1 methanol: acetic acid for 10 min on ice. Metaphase spreads were then prepared by dropping cells onto methanol-washed positively charged slides. Slides were stained with Giemsa stain and sealed. Images of 100–150 metaphase spreads from 4–6 individual animals were captured using ×100 objective and chromosomal breaks and radial chromosomes were visually counted using FIJI software.

### Fork protection assay

Fork protection assay of single-molecule DNA fibers was performed similarly as in published protocols^20,34^. Briefly, splenic B cells or sorted LSKs were cultured for 3 days then replicating DNA was labeled by a 30 minute pulse with 50 µM IdU (Sigma) followed by a 30 minute pulse with 250 µM CldU (Sigma). Cells were washed then treated with HU (Sigma) (4 mM for spleenic B cells or 2 mM for BM HSPCs) for 3 h. Cells were lysed directly on positively charged microscope slides and then tipped to 30° to spread single DNA fibers. Slides were subsequently fixed and DNA denatured and neutralized. Slides were incubated with anti-CIdU (rat, abcam) and IdU (mouse, BD Biosciences) primary followed by goat anti-rat AF488 and goat anti-mouse AF568 (Invitrogen) secondary antibodies. DNA fibers were captured using ×100 or ×60 objective on a Nikon Eclipse 80*i* fluorescent microscope and quantified using FIJI software.

### Statistical analysis

For all cell culture, CFC assay and BMT assays, two-tailed Student’s *t*-tests were performed. Graphs are presented as mean ± SEM. For metaphase analysis, statistical comparisons were made using one-tailed ANOVA and Tukey’s post test in PRISM software. For DNA fiber ratios in the DNA fiber fork protection assays, Kruskal–Wallis one-way analysis of variance test was used for nonparametric data, and comparisons between individual groups were calculated using Dunn’s multiple comparison post test in PRISM software. A *p* value of less than 0.05 was considered statistically significant.

## Electronic supplementary material

Supplementary Information

## Data Availability

All relevant data that support the findings of this study are available from the corresponding author upon reasonable request.
