# Supplementary material for: Lnk/Sh2b3 deficiency restores hematopoietic stem cell function and genome integrity in Fancd2 deficient Fanconi anemia
Source: Nat Commun. 2018 Sep 25;9:3915. doi: 10.1038/s41467-018-06380-1 (PMC6156422; doi:10.1038/s41467-018-06380-1)
Supplement: Supplementary file 1 — Supplementary Information [file 41467_2018_6380_MOESM1_ESM.pdf]

Supplemental information for

Lnk/Sh2b3 Deficiency Restores Hematopoietic Stem Cell Function and Genome Integrity in *Fancd2* Deficient Fanconi Anemia

Joanna Balcerek *et al.*

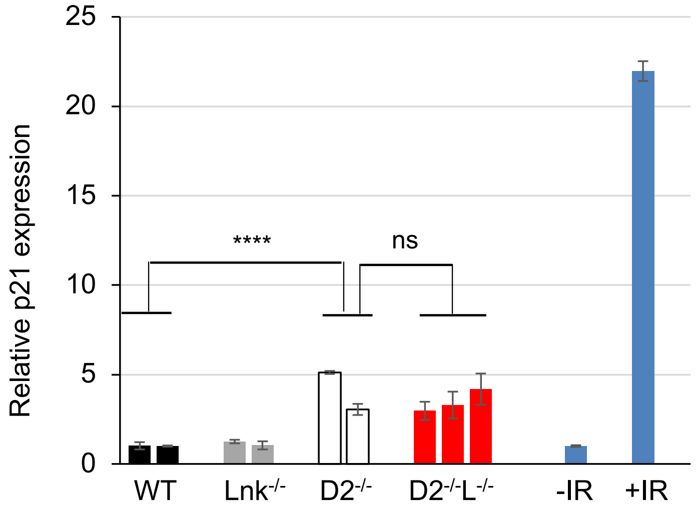

Supplemental Figure 1: ***Fancd2*** **deficiency modestly induces p53 and** ***Lnk*** **deficiency does not mitigate p53 activation at the steady-state** ***in vivo***. LSK cells were sorted from WT, Fancd2$^{-/-}$ (D2$^{-/-}$), Lnk$^{-/-}$ (L$^{-/-}$) and FancD2$^{-/-}$;Lnk$^{-/-}$ (D2$^{-/-}$L$^{-/-}$) mice. The expression of p53 target gene p21 was measured by qRT-PCR. The relative p21 expression was first normalized against Ub-C internal control followed by normalization to WT levels. One hour post with or without irradiation (+IR and -IR) are used as controls. Each bar represents individual animal and mean±SE of triplicates is shown. **** $p<0.0001$, determined by two-tailed student's t-test are shown. ns: not significant.

LNK Blot

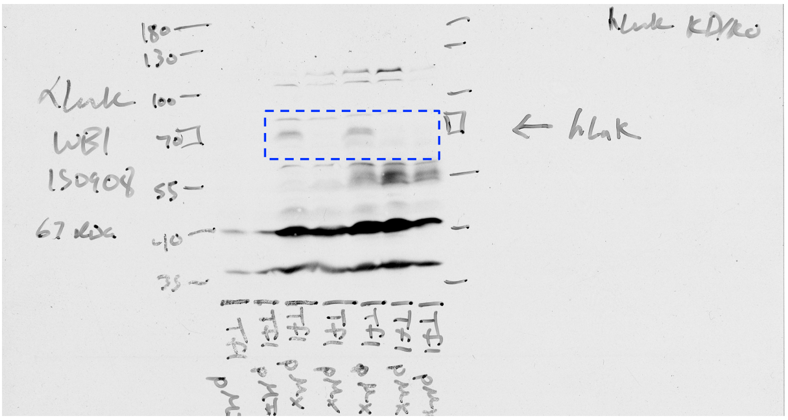

Actin Blot

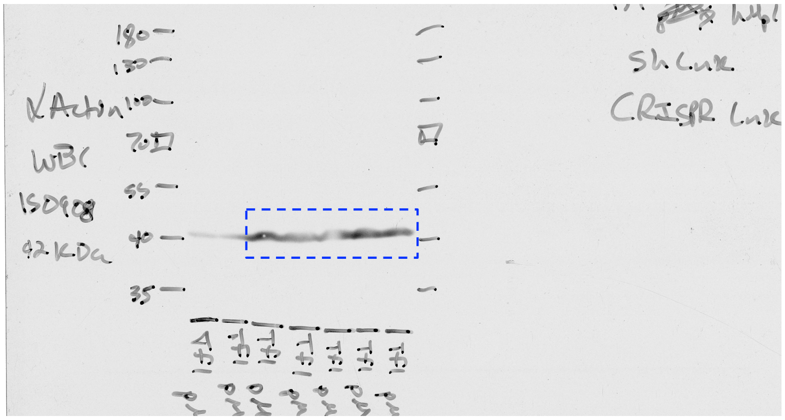

FANCD2 Blot

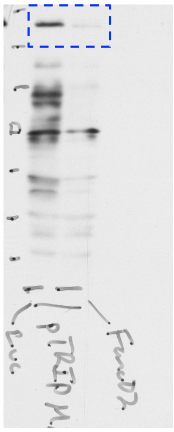

Actin Blot

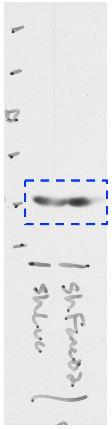

Supplemental Figure 2: Uncropped scans for Western blots sown in Figure 7C.
